# Fault Detection of Bearing Systems through EEMD and Optimization Algorithm

**DOI:** 10.3390/s17112477

**Published:** 2017-10-28

**Authors:** Dong-Han Lee, Jong-Hyo Ahn, Bong-Hwan Koh

**Affiliations:** Department of Mechanical, Robotics and Energy Engineering, Dongguk University-Seoul, 30 Pildong-ro 1 gil, Jung-gu, Seoul 100-715, Korea; micro89@hanmail.net (D.-H.L.); dkswhd83@gmail.com (J.-H.A.)

**Keywords:** EEMD, Isomap, PSO, fault detection, feature extraction

## Abstract

This study proposes a fault detection and diagnosis method for bearing systems using ensemble empirical mode decomposition (EEMD) based feature extraction, in conjunction with particle swarm optimization (PSO), principal component analysis (PCA), and Isomap. First, a mathematical model is assumed to generate vibration signals from damaged bearing components, such as the inner-race, outer-race, and rolling elements. The process of decomposing vibration signals into intrinsic mode functions (IMFs) and extracting statistical features is introduced to develop a damage-sensitive parameter vector. Finally, PCA and Isomap algorithm are used to classify and visualize this parameter vector, to separate damage characteristics from healthy bearing components. Moreover, the PSO-based optimization algorithm improves the classification performance by selecting proper weightings for the parameter vector, to maximize the visualization effect of separating and grouping of parameter vectors in three-dimensional space.

## 1. Introduction

A bearing system is one of the most crucial components in rotating machinery. Preemptive fault detection and diagnosis algorithms are required to prevent unpredicted malfunctions or system failure. Most of the methods monitor the deviation of dynamic properties of the bearing system under operating conditions. By continuously measuring the changes of the bearing components over time, the dynamic characteristics of a fault-sensitive feature are extracted, and the current status of system integrity is determined through various statistical techniques. Through this series of processes, it is possible to minimize the probability of catastrophic accidents caused by unpredicted defects to structures, and to improve the reliability of normal operation of the rotating system. Furthermore, continuous maintenance is essential, since the bearing system is subject to continuous load, and defects due to fatigue or vibration are likely to occur alongside it. Initial micro-defects in the bearing system grow and break down, which can cause fatal damage or accidents to the entire system, including rotating equipment. In general, bearing defects occur in the rolling element, outer ring, and inner ring, resulting in repetitive micro-collisions that interfere with smooth rotational motion. It is known that the vibration signals generated from defect-free rolling bearings generally follow a Gaussian distribution [1]. In the case of a damaged bearing, the defective part with a non-smooth surface collides with its counterpart, and the repetitive shock wave appears as a cyclostationary signal. Since the characteristics of the bearing signal generated by different types and locations of bearing system are good indicators of remaining service life, techniques for analyzing the vibration signals have been widely explored in various fields [2]. Many bearing condition monitoring techniques combine various signal processing techniques for separating features related to defects, and machine learning methods for extracting and classifying the characteristics of the disassembled signal.

Nikolaou [3] proposed a technique for analyzing a defective signal from bearing vibration measurements using wavelet packet decomposition, and extraction of the dominant frequency components from the defective bearing. Lou [4] decomposed the bearing signal through wavelet transforms, and characterized the standard deviation of the decomposed signal, to identify defects, by developing a neuro-fuzzy model. Konar [5] conducted a study of the bearing defects of the induction motor, which is a type of AC motor. The study performed continuous wavelet transform by exploiting Morlet wavelets and Daubechies wavelets, and obtained wavelet coefficients by a support vector machine (SVM) and an artificial neural network (ANN). A method of detecting defects through time-frequency decomposition, such as the Hilbert-Huang transform (HHT), has also been proposed [6]. Moreover, techniques for decomposing signals using empirical mode decomposition (EMD), an intermediate process of HHT, have been used. Junsheng [7,8] also employed decomposition of the bearing signal through EMD, created the autoregressive (AR) model using the decomposed signals, and identified the type of bearing defects by separating the coefficients of the AR model. In addition, EMD demodulation technique was used to extract instantaneous frequency amplitudes, and to identify the bearing defect. Dejie [9] performed wavelet decomposition to extract the features due to bearing defects. The study extracted the wavelet coefficients of high levels, and reconstructed the signals. In addition, mode mixing, which is one of the side effects of the EMD, can be removed through Ensemble Empirical Mode Decomposition (EEMD). Specifically, EEMD can be used to prevent mode-mixing, so that the degradation of the damage detection performance, in which an intrinsic mode function (IMF) signal is projected onto another IMF, is reduced. Lei et al. [10] selected the IMF based on the kurtosis of the signal decomposed by EEMD, and employed the wavelet neural networks (WNN) method using wavelets and artificial neural networks to detect the defects of roller bearings in railway vehicles, respectively. Zhang [11] introduced a damage detection and classification study using EEMD and multi-class SVM. Also, Wang [12] analyzed EEMD signals of bearing system through tunable Q-factor wavelet transform (TQWT). Zhao [13] proposed SVM-based damage detection using EEMD and multi-scale fuzzy entropy. Jiang [14] applied Improved Ensemble empirical mode decomposition (IEEMD), which is an upgraded version of EEMD to tackle the noise issue, to decompose bearing signals and extract statistical features from the signals, to distinguish bearing defects using back propagation of neural networks. As can be seen from the literature, monitoring of bearing system is mostly confined to the adaptation of signal processing techniques. It may be worthwhile to incorporate computational intelligence or data-based learning approach that enhances classification performance for reliable decision-making processes. 

In this study, we investigate signal processing methods in conjunction with an optimization algorithm to isolate the damage of a bearing system, and identify the types of defects. The typical signal of bearing failure appears as a mixture of periodic shock waves superimposed on the noise from normal bearings. In order to distinguish the characteristics of the damaged and healthy bearing signals, a technique for resolving the fault signal needs to be applied first. The time-frequency decomposer EEMD was used to decompose the acceleration measurement of a bearing with defects. Specifically, EEMD is applied to segments, which are divided into uniform time intervals, and the statistical features of each signal are extracted to manifest the characteristics of the bearing condition. This process generates the EEMD-based feature vector of the time segment signal, which is used for defect detection. The statistical properties of time-series data, such as standard deviation, root mean square (RMS), kurtosis, zero-crossing rate, and entropy, have different ranges of values. Thus, the characteristic values having different ranges vary in sensitivity to defects, because the weights of defects are different from each other. In order to prevent bias of weightings in parameters, we normalized the same characteristic values to generate equivalent vectors. The feature vectors are then visualized using two-dimensional reduction techniques, PCA, and Isomap.

It is expected that the feature vectors having the same defect case are located at a distance adjacent to each other to form one defect group. However, depending on the nature of defect, the generated feature vector may have both defect-sensitive and insensitive characteristics and weightings, which may deteriorate the classification performance. In order to solve this problem, particle swarm optimization [15], which is one of the most reliable optimization algorithms, was used to determine a linear weight to the feature vector, and to optimize its coefficients. We used the Dunn index [16,17], which is typically used in classification analysis, to indicate whether the objective function of PSO is well distinguished from other groups. The dimension of the optimized results was reduced by two to visualize the classification. PCA, Isomap, and non-optimized results were all compared for discussion. Finally, the aforementioned techniques that have been applied to a mathematical model for simulating bearing defects were verified using actual data, i.e., experimentally measured defective bearing signals.

## 2. Background Theories

### 2.1. Empirical Mode Decomposition

Huang proposed a systematic data processing algorithm, or EMD, that is specifically designed to decompose nonlinear and non-uniform time-domain signals [18]. Through the EMD process, every time-domain data can be divided in terms of zero-crossings, and fully reconstructed as a linear combination of multiple functions or IMFs. The general procedure for decomposing EMD into IMF is as follows:

1. Find the local maxima and local minima of the signal x(t).
2. Connect the local maximum points as a spline to develop the upper envelope. Likewise, connect the local minimum points to obtain the lower envelope.
3. Obtain the average value m(t) of the upper envelope and lower envelope from Step 2.
4. Subtract the average value m(t) from the signal x(t) to obtain the value $h_{1}\left(t\right)$.
5. The value $h_{1}\left(t\right)$ becomes the first IMF of the signal, if it satisfies the following two conditions: (1) the difference between the number of maxima and zero crossings is zero or one; (2) The average of the upper and lower brackets is zero. If not, repeat Steps [1–5], until the $h_{1}\left(t\right)$ satisfies both conditions.
6. Having finished *k* iterations, $h_{1\left(k-1\right)}\left(t\right)-m_{1k}\left(t\right)=h_{1k}\left(t\right)$ or $c_{1}\left(t\right)=h_{1k}\left(t\right)$. If $c_{1}\left(t\right)$ satisfies the above conditions of IMF, then separate $c_{1}\left(t\right)$ by subtracting it from the original signal x(t), i.e., $r_{1}\left(t\right)=x\left(t\right)-c_{1}\left(t\right)$.
7. The above process is repeated, until the remaining $r_{i}\left(t\right)$ has less than two extremes.

Finally, when all of the *n*-th IMFs are obtained, the original signal x(t) will appear as shown in Equation (1) below, resulting in EMD:

(1) $$x\left(t\right)=\overset{n}{\underset{i=1}{\sum }}c_{i}\left(t\right)+r\left(t\right)$$

### 2.2. EEMD and Feature Extraction

**Figure 1:**
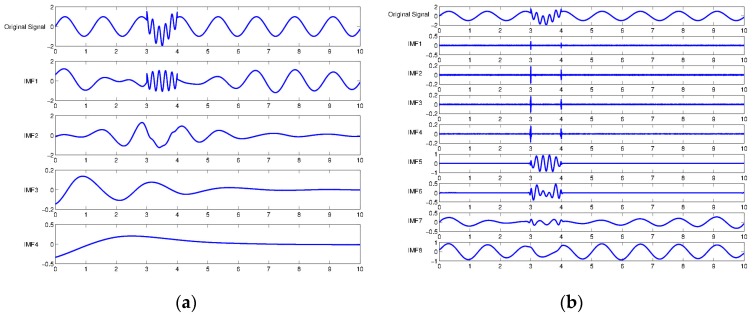
Exemplary illustration of mode mixing problem in empirical mode decomposition (EMD) and ensemble empirical mode decomposition (EEMD): (**a**) EMD; and (**b**) EEMD.

It is widely known that EMD has suffered from a mode mixing problem, such that the individual IMFs tend to be blended together [19,20]. Obviously, EEMD is proposed to tackle the issue of mode mixing by inserting Gaussian noise into the original signal before proceeding to EMD, and iterating the process of averaging out of IMFs to eliminate mixed modes [21]. In this section, we compared the EMD and EEMD by showing how the sinusoidal component is isolated in IMF. Figure 1 shows that in the first IMF of the EMD result, sine waves of different frequencies were mixed. Because of the presence of mode mixing in low IMFs, higher IMFs also failed to decompose two different sinusoidal signals. One the other hand, the EEMD successfully separated the high frequency portion in the low IMFs, so that high-frequency and low-frequency signals were clearly isolated in IMF5 and IMF7, respectively.

While recognizing the IMFs of EEMD from bearing signals is one of the convenient representations of bearing conditions, it is impractical to use the IMF without extracting damage-sensitive features. Here, we considered several statistical parameters, such as standard deviation, root mean square (RMS), kurtosis, zero-crossing rate, and entropy from each IMF, to evaluate the condition before and after the presence of defect in the bearing system. Table 1 shows such parameters that were extracted from the IMFs of EEMD to develop a feature vector for both healthy and damaged bearings. To reduce deviations towards the sensitivity of the bearing defect, all the elements of the feature vector have been equally weighted and initially normalized.

### 2.3. Isomap

For dimensionality reduction, Isomap algorithm performs nonlinear data embedding through multi-dimensional scaling (MDS), which successfully upholds distances between data points in low-dimensional space [22]. This isometric feature mapping approach exploits global characteristics of geodesic distances of each data point, and computes piece-wise Euclidean distances between neighbor data points. In other words, Isomap inherently creates connecting graphs between a nonlinearly distributed data set based on nearest neighbors from specific data. Using Dijkstra’s algorithm [23] and the Floyd-Warshall algorithm [24,25], Isomap calculates the shortest distance among the connecting nodes of the graph, so that a high-dimensional data structure iteratively transfers into a reduced dimensional space through embedding processes. The first step of the Isomap algorithm is to identify the *k*-nearest neighbors of each point, in which neighborhood relations are represented by a graph G. Each data point is connected to its nearest neighbors by edges. In the second step, the algorithm estimates geodesic distance between all pairs of points in the data. The Floyd-Warshall algorithm is employed to find the matrix of the graph containing the shortest path distances between all pairs of points in G. Finally, classical MDS is performed, to create lower dimensional embedding of the data with the shortest path in Euclidean space.

### 2.4. Particle Swarm Optimization (PSO)

The underlying concept of PSO is adopted from observation of the collective performance of animal groups and their individuals towards the task of foraging food or avoiding predators [15]. Since its appearance in the early 2000s, PSO has become one of the most widely used optimization algorithms. In PSO, a particle or an individual in a group shares the information of both local and global optima with other members in moving around the search space, in which every particle is randomly assigned its position and velocity. Once iteration begins, all the particles are dispersed to search for optimal points by updating their current positions and velocities at every time step, based on the contest result of their function values from the previous step. The search process is formally illustrated by Equations (2) and (3):

(2) $$v_{i}^{t+1}=w_{i}^{t}v_{i}^{t}+c_{1}r_{1}\left(p_{i}^{t}-x_{i}^{t}\right)+c_{2}r_{2}\left(p_{i}^{all}-x_{i}^{t}\right)$$

(3) $$x_{i}^{t+1}=x_{i}^{t}+v_{i}^{t}$$

Here, the current position and velocity of the particle are denoted by $x_{i}^{t}$ and $v_{i}^{t}$, respectively. They are used to update the position of the particle in the next step or $x_{i}^{t+1}$, as shown in Equation (2). The updated velocity $v_{i}^{t+1}$ is derived by considering the current velocity ($v_{i}^{t}$), the weighted difference between the optimal positions of local and global ($p_{i}^{t}$), along with the current positions ($x_{i}^{t}$). Note that $r_{1}$ and $r_{2}$ are randomly varied between zero and unity, to impose an unbiased balance of the search direction towards the local and global optimum. Also, the level of influence or learning factors $c_{1}$ and $c_{2}$ of local and global optima are usually set to 2, to achieve moderate convergence speed and relatively high accuracy. The coefficient $w_{i}^{t}$ regulates the search speed of particles, so that a higher $w_{i}^{t}$ allows extensive jumping of search ranges, while a smaller value limits the search boundary to the vicinity of the current position. Thus, a strategy of initially setting the coefficient to a large value for extensive search is recommended in the early stage. Then, we slowly let it decay to a small value for detail or local scrutiny, as the search process approaches its conclusion. It is known that for reliable search performance in PSO, Equation (4) provides an appropriate value of $w_{i}^{t}$ in terms of the maximum iteration number $iter_{max}$ and current step number $iter_{t}$.

(4) $$w_{i}^{t}=\frac{iter_{max}-iter_{t}}{iter_{max}}$$

## 3. Simulation of Bearing Response and Fault Detection Results

Because of the cyclic nature of impacts between the defect and the surface of bearing elements, vibration responses from different damage locations, such as the inner-race, outer-race, and rolling element, exhibit a unique pattern of spectra. There have been many dynamic models to represent the response of defects in rolling bearing systems. Among them, Tao [26] suggested an improved simulation model of McFadden and Smith [27] to produce responses of bearing defects in the inner-race, outer-race, and rolling elements as $x_{inner}$, $x_{outer}$ and $x_{rolling}$, respectively (see Equations (5) and (6)).

(5) $$x_{inner}\left(t\right)=\overset{\infty }{\underset{k=-\infty }{\sum }}A\left(kT_{d}\right)e^{-\xi \left(t-kT_{d}\right)}\sin\left(2\pi f_{s}\left(t-kT_{d}\right)+\phi_{k1}\right)U\left(t-kT_{d}\right)$$

(6) $$x_{outer}\left(t\right)=\overset{\infty }{\underset{-\infty }{\sum }}e^{-\xi \left(t-kT_{d}\right)}\sin\left(2\pi f_{s}\left(t-kT_{d}-\frac{T_{d}}{2}\right)+\phi_{k2}\right)U\left(t-kT_{d}-\frac{T_{d}}{2}\right)$$

Here, $f_{s}$ denotes the resonance frequency of the overall system, $f_{r}$ is the shaft rotation frequency, U(t) indicates a unit step function, and A(t) shows the amplitude modulation function, in which the inner-race defect can be expressed as $A\left(t\right)=A_{0}\;max\left(cos\left(2\pi f_{r}t\right),0\right)cos\left(2\pi f_{r}t\right)$. Also, note that as the rolling element collides with the defect surface with the same force, A(t) remains constant for the case of defects occurring in the outer-race. The rotating frequency of the shaft is defined as $f_{r}$. Table 2 shows that the defect frequency of the inner-race $f_{di}$, outer-race $f_{do}$, rolling element $f_{dr}$, and damping factor ξ are also considered for the simulation of vibration response of the bearing system. On the other hand, when the component of the rolling element becomes defective, a portion of the defect collides with both surfaces of the inner and outer ring to create repetitive impact signals. Thus, the combination of the defect signal becomes the summation of inner $x_{inner}\left(t\right)$ and outer ring part, $x_{outer}\left(t\right)$. At this time, since the magnitude of the defect signal generated in the inner ring is smaller than that of the outer ring, the ratio coefficient μ is smaller than one. Therefore, the defect signal of rolling element $x_{rolling}\left(t\right)$ can be obtained as shown in Equation (7):

(7) $$x_{rolling}\left(t\right)=x_{outer}\left(t\right)+\mu \;x_{inner}\left(t\right)$$

**Figure 2:**
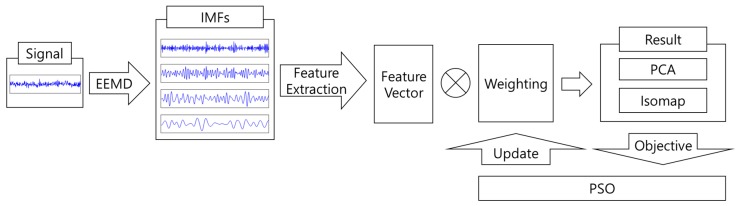
A schematic diagram of overall process for bearing fault detection.

**Figure 3:**
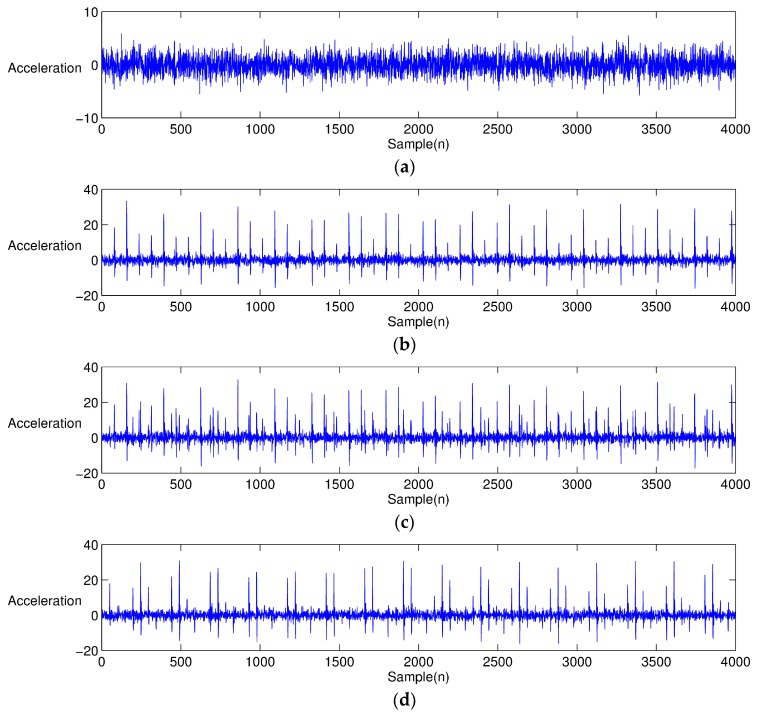
Simulation signals of a bearing system: (**a**) healthy condition (**b**) inner-race defect (**c**) outer-race defect, and (**d**) rolling element defect.

**Figure 4:**
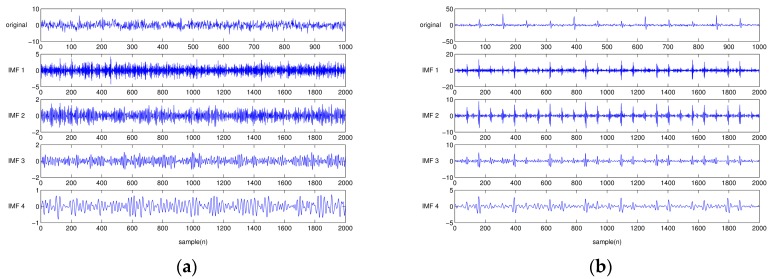

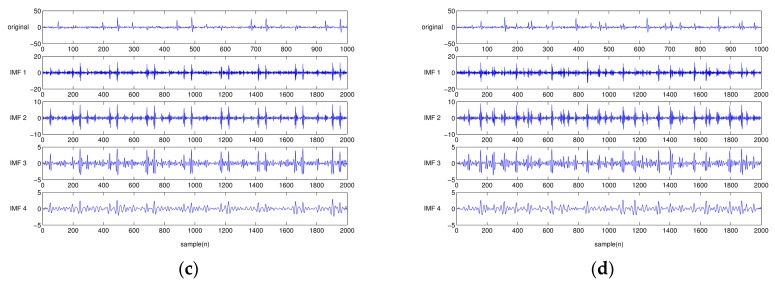
The first four EEMD-decomposed intrinsic mode functions (IMFs) of simulated time-series data: (**a**) healthy bearing model; (**b**) inner-race damaged bearing model; (**c**) outer-race damaged bearing model; and (**d**) rolling-element damaged bearing model.

In this study, to verify the technique of bearing fault detection, the defect signal of the bearing was first generated using the Equation (7). The overall process of bearing fault detection and classification is illustrated in Figure 2. Gaussian distribution noise is mixed with the steady-state bearing signal having a defective bearing component. Table 2 shows the constant values of the equation used for generating the bearing signal:

Figure 3 shows the vibration signals of different bearing conditions reproduced from the previous Equations (5)–(7) and Table 2. Figure 3a shows the normal bearing condition, for which there is no shock signal, but a noise with Gaussian distribution. On the other hand, Figure 3b–d shows that bearings having different defect types produce repeated local-spikes. Figure 4 illustrates the EEMD-performed results from Figure 3. Note that only the first four IMFs are presented for simple comparison. The characteristics of the individual IMF signal differ from each other, depending on the type of bearing defect. For the healthy bearing condition, each IMF is similar to that obtained by simply decomposing a white noise. Obviously, it is difficult to find the characteristic portion of impacts in EEMD from the healthy bearing signal (see Figure 4a). However, in the case of EEMD performed on the bearing signal with a defect, the impact nature due to the defect is mostly dominant in the first three IMFs. When a bearing element rotates, the impulse is repeated at a predetermined interval. The signal characteristics, such as the interval at which the impact occurs, and the intensity of the generated signal, are unique, depending on the type of defect: an inner ring defect, outer-ring defect, or rolling element defect.

Again, we employed EEMD to decompose the time-series of the bearing signals. For each damage case, 16 time-segments having data points of 0.125 s duration were decomposed into eight IMFs. In order to develop a feature vector, five parameters, such as the kurtosis, root mean square, standard deviation, zero-crossing rate, and entropy, are extracted from each IMF. Thus, an individual time-segment produced a vector having 40 feature elements. In summary, 16 time-segments were collected from each of the three cases of bearing defects and one healthy condition. All of the parameters were normalized, to reduce systematic irregularities and biases. Because each segment represented a state of the current bearing condition, it could be visualized as a point in three-dimensional space through a PCA or Isomap-based dimensional reduction.

Although the feature-extracted parameter vector itself represented a unique signature of a bearing condition, each element of those parameters may have a different level of contribution in classifying damage types or categories. Thus, it is reasonable to assign a proper weight to each element of the parameter vector to achieve the best classification performance. We can find optimal sets of parameter weights that balance both goals, of maximizing the effect of separation from different defect types, and by minimizing the Euclidian distance among the points (parameter vectors), from the group of identical bearing defects. Here, a point or parameter vector represents a single state of bearing condition in multi-dimensional space from a segment of time-series data.

This study employed the PSO algorithm to find weightings for the parameter vector. Having developed parameter vectors, PSO iteratively searched for the best set of weights in all 40 feature-elements, while minimizing the Euclidian distances among points, and maximizing the separations between damage groups. Hence, the objective function Q to determine parameter weightings was the Dunn index (Equation (8)) for assessing the performance of grouping in classification problems [16]:

(8) $$Q=\frac{\underset{1\leq i<j\leq m}{\min}d\left(i,j\right)}{\underset{1\leq k\leq m}{\max}d_{k}^{\prime }}$$

**Figure 5:**
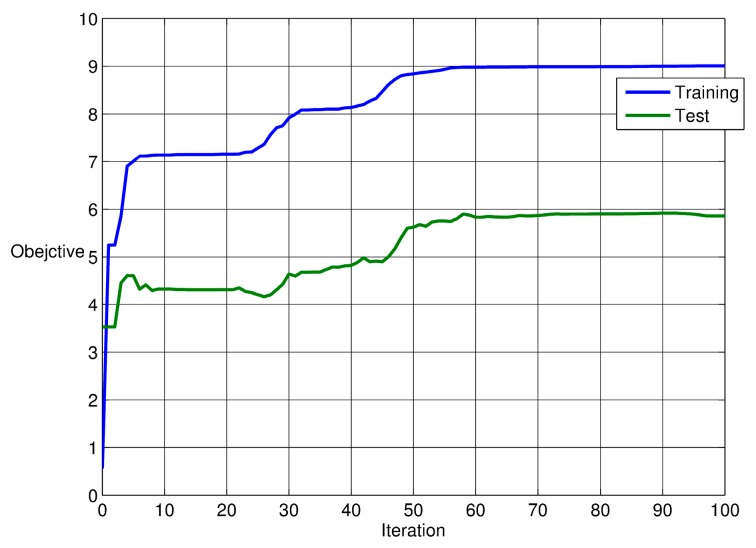
Particle Swarm Optimization (PSO)-based optimization result of the weightings of parameter vectors extracted from time-segments of various damaged bearing models.

Here, d(i,j) is the distance between the *i*-th and *j*-th group in a total *m* number of groups. The largest distance between any members in the *k*-th group is denoted as $d_{k}^{\prime }$. Thus, a larger value of Q implies that each group becomes more successfully separated in multi-dimensional space. Obviously, maximizing the Q through PSO provides an effective measure for quantifying the classification performance in terms of bearing defect category. Figure 5 illustrates convergence curves of the PSO searching process for a set of weights having maximum Q. We tried several independent PSO runs to seek a best convergence result, and used the weights as a trained data. Figure 5 shows the best training run and test run. The figure shows that the value Q increased from 0.56 to 9.01 in the training case. Similarly, the test case results in a relatively small increase of Q (from 3.53 to 5.86). This trend of variation contributes to the fact that group-to-group or d(i,j) remains the same, while the point-to-point distance or $d_{k}^{\prime }$ increases more significantly. Note that half of the total data sets were used for optimization *training*, while the other half were used for *testing* the PCA classification.

**Figure 6:**
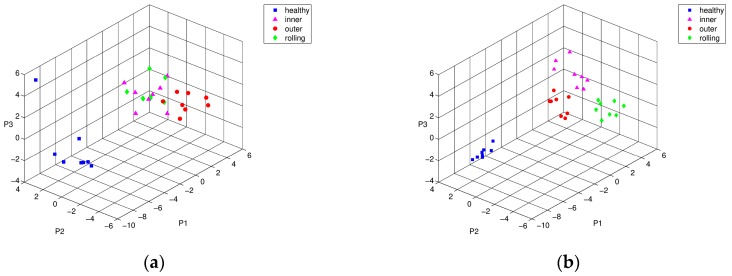
Principal component analysis (PCA) representation of training characteristic vectors from simulated bearing model: conditions of intact (■), inner-race defect (▲), outer-race defect (●), and rolling-element defect (♦), (**a**) before, and (**b**) after PSO optimization.

**Figure 7:**
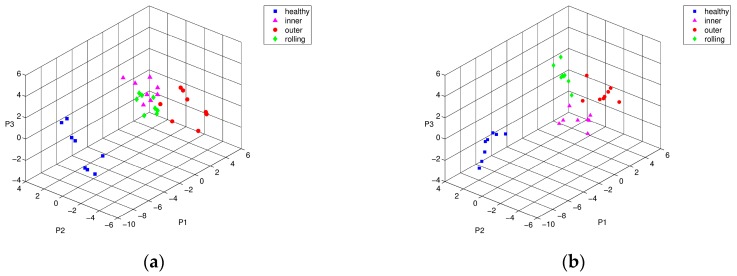
PCA representation of testing characteristic vectors from simulated bearing model: conditions of intact (■), inner-race defect (▲), outer-race defect (●), and rolling-element defect (♦), (**a**) before, and (**b**) after PSO optimization.

Figure 6 shows the PCA-based localization results for comparison; before and after performance of the weight-optimization of feature vectors using the training time-series segments towards different damage locations, i.e., inner, outer-race, and rolling elements. Apparently, the first three principal components of PCA show that feature vectors of inner-race and rolling element damage are mostly inseparable in three-dimensional space, as shown in Figure 6a. On the other hand, weight-optimized feature vectors show clear distinction and separation between the two groups of defects (see Figure 6b). Having finished training, we applied the weight-optimized parameter vectors to the test data set. Figure 7 shows the comparison of the PCA results of the test data using the trained parameter vectors. Figure 7a shows that the inner ring defect and the rolling element defect are partially mixed in a reduced three-dimensional space. The figure was obtained by performing PCA that had not been subjected to the optimization process, the elements of each group of defects were widespread, and there was a risk that the type of defects may have been misleading. However, Figure 7b shows that the feature vectors optimized by PSO were tightly grouped together by the same defect, so that it was easier to visually distinguish the inner ring defects and rolling element defects. 

**Figure 8:**
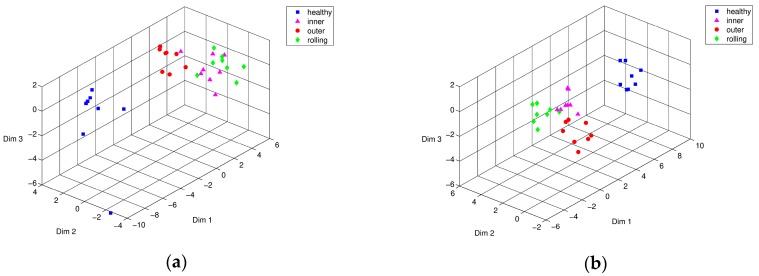
Isomap representation of training characteristic vectors from simulated bearing model: conditions of intact (■), inner-race defect (▲), outer-race defect (●), and rolling-element defect (♦), (**a**) before, and (**b**) after PSO optimization.

**Figure 9:**
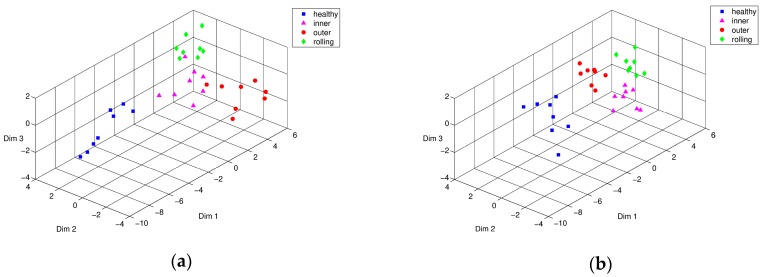
Isomap representation of testing characteristic vectors from simulated bearing model: conditions of intact (■), inner-race defect (▲), outer-race defect (●), and rolling-element defect (♦), (**a**) before, and (**b**) after PSO optimization.

Likewise, Figure 8 and Figure 9 shows the Isomap classification results of training and testing data sets, respectively. Figure 8a shows that the boundaries of the inner-race and rolling element defects partially overlapped. Obviously, this training process enhanced the performance of Isomap representation in different types of bearing defect, as shown in Figure 8b. Figure 9 shows the visualization result of Isomap classification in three-dimensional space using testing data sets. Similar to the PCA results without the weight optimization process, the inner ring defect and the rolling element defect were positioned within the same group adjacent to each other (see Figure 9a). Figure 9b shows the result of optimization through PSO, wherein the inner-race defect and the rolling element defect were clearly distinguishable from each other. Overall for both cases, PSO-based weight optimization significantly enhanced discrimination of the different types of bearing defects.

It also seems that the performance of Isomap representation with PSO optimization equaled or slightly exceeded that of PCA with PSO results. Comparing the Isomap results with those of PCA showed that the distance between points within the same defect group increased, but the distance between the other defect groups has increased even more, making it much easier to distinguish them from each other. This is because the Isomap algorithm possesses the property of keeping the phase of the characteristic vector in the multidimensional space as much as possible, even at low dimensions (two- or three-dimensions). Thus, this advantage is particularly useful for distinguishing defective groups, since they provide an enhanced visualization when projecting high dimensional data onto a low dimensional space. 

## 4. Experimental Procedure

**Figure 10:**
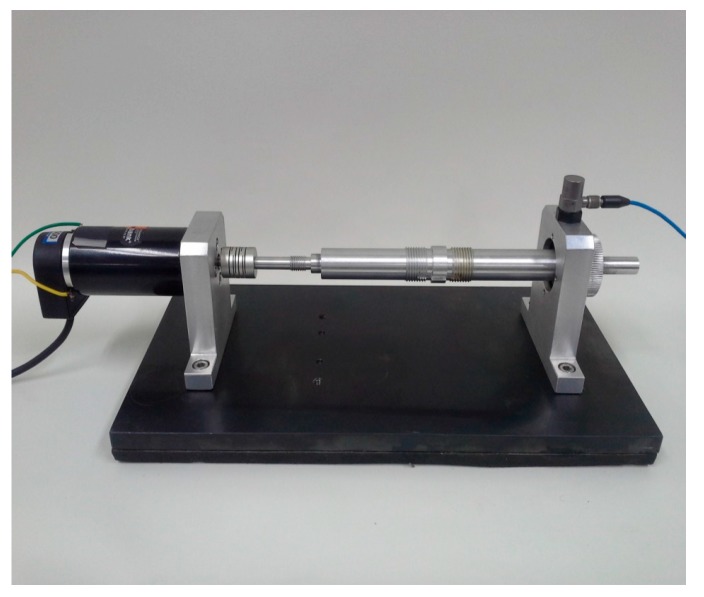
Experimental setup for a roller bearing system.

This section illustrates the experimental validation of the aforementioned damage monitoring technique using a motor-driven shaft that is supported by roller-type bearings. Figure 10 shows that the vibration response of the system can be recorded through an accelerometer and pertinent data acquisition system. To suppress external disturbances, a mechanical coupling was positioned in the middle of the shaft. An optical encoder embedded in the brushless motor controlled the rotational speed to maintain 2400 rpm. An accelerometer (PCB 352C33) having 100 mV/g is mounted on top of the housing, to measure the vertical movement of the bearing system. Data acquisition hardware of PXI-1042Q (National Instruments) was used for data collection, with a sampling frequency of 10 kHz. 

**Figure 11:**
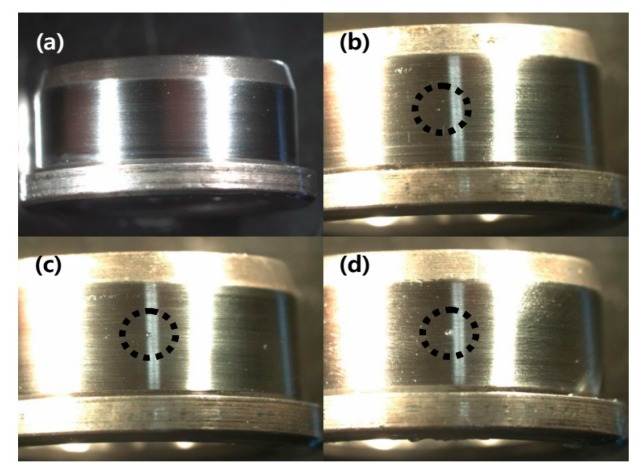
Sampled pictures of different damage levels: (**a**) healthy bearing condition (H); (**b**) low-level dent-type defect on a bearing inner-race (D15); (**c**) middle-level dent-type defect on a bearing inner-race (D30); and (**d**) high-level dent-type defect on a bearing inner-race (D45).

**Figure 12:**
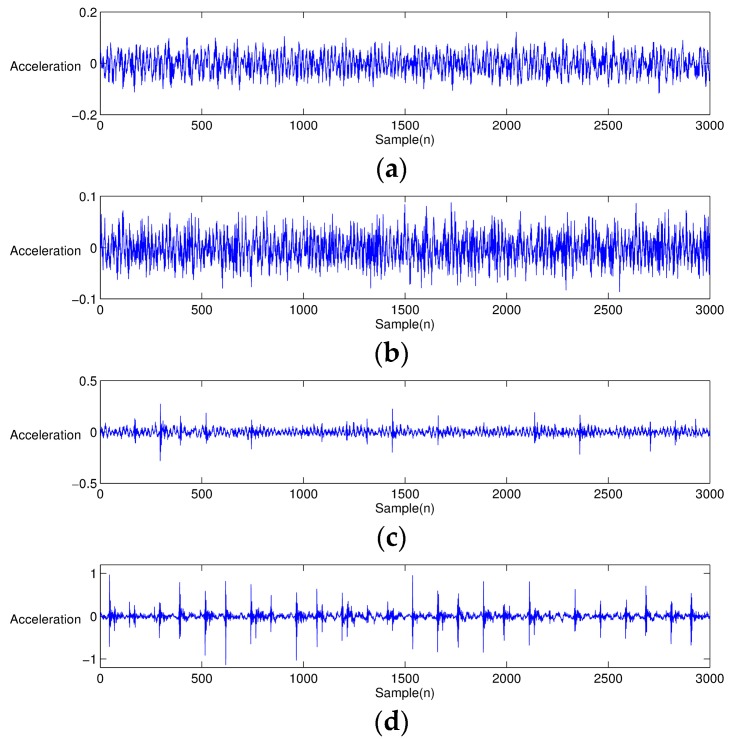
Acceleration measurements collected from the bearing test-bed: (**a**) healthy bearing condition (H); (**b**) low-level damage (D15); (**c**) middle-level damage (D30); and (**d**) high-level damage (D45).

**Figure 13:**
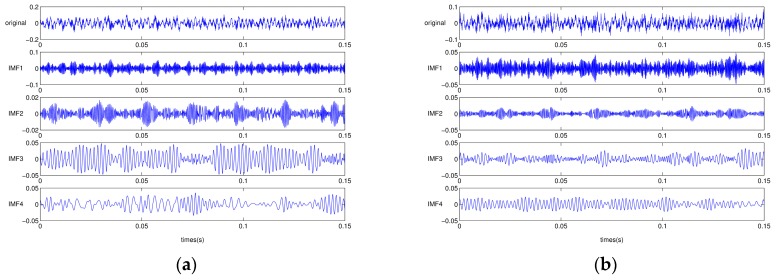

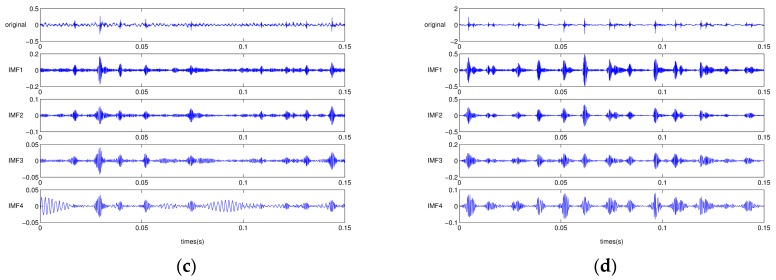
First four EEMD-decomposed IMFs of actual vibration signals: (**a**) healthy bearing condition (H); (**b**) low-level damage (D15); (**c**) middle-level damage (D30); and (**d**) high-level damage (D45).

The type of roller bearings used in the test was SKF NJ 202 ECP. Here, we chose an indent-style notch damage to imitate a partial spalling condition of the bearing surface. The indentation damage was easy to implement at a large quantity, and uniformly replicable for creating the identical damage severity of test bearings. For this indentation, we used HR-500, a diamond-tipped hardness test machine. To make three different levels of indentation defects, the HR-500 applied (15, 30, and 45) N forces to the surface of the inner-race of test bearings. Figure 11a–d shows the size of indentation defects on the inner-race created by (15, 30, and 45) N forces, respectively, applied by HR-500. Figure 11a shows the condition of healthy bearing with no defect. Hereinafter, the severity of defect was denoted as D15, D30, D45, and H for intact bearing. Figure 12 shows the time-series response of H, D15, D30 and D45. From the figure, both D30 and D45 show clear visual distinction caused by the presence of the bearing defect. Note that the case of D15 barely revealed defect-induced spike-type responses. Figure 13a–d depicts the EEMD of one healthy and three different damaged bearing signals. For meaningful comparison, the figures are only shown up to the first eight IMFs. Similar to the time-series responses, the corresponding IMFs of H and D15 show few distinctions.

## 5. Results and Discussions

**Figure 14:**
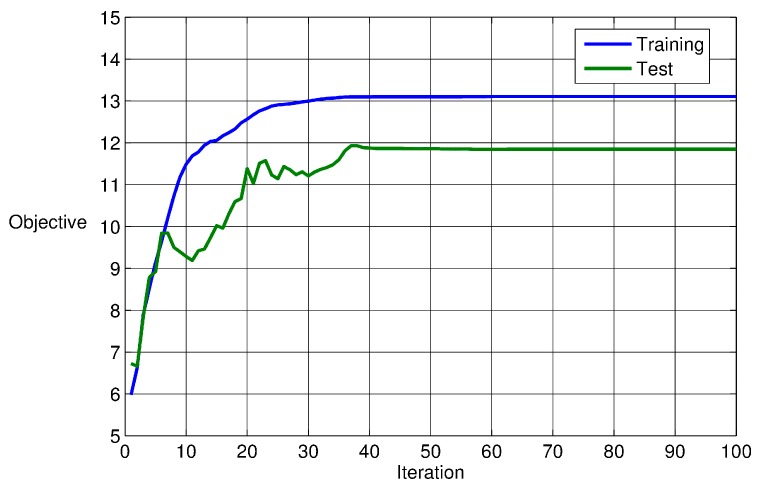
PSO-based optimization results of the weightings of parameter vectors extracted from various time-segments having an inner-race defect in the bearing testbed.

**Figure 15:**
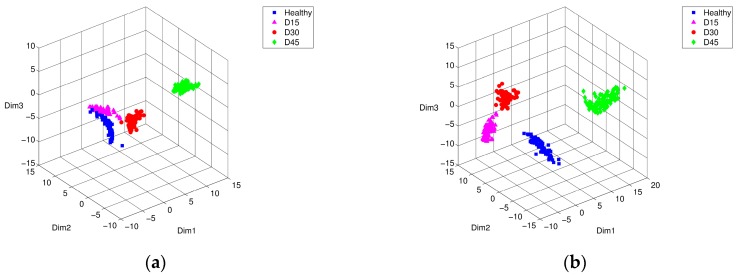
Isomap representation of the training characteristic vectors from a bearing testbed: conditions of Healthy (■), D15 (▲), D30 (●), and D45 (♦), (**a**) before, and (**b**) after PSO optimization.

This section discusses the results of employing PSO to find the optimized weights of damage-sensitive feature vectors using the bearing testing data. We trained independent data sets of time-series of the bearing system to find optimized weights for the feature vector. Having finished training, the weights were applied to the new data set to validate the performance and feasibility of the proposed method. Compared to the case of all-unity-weights, the converged set of weights from training data showed better performance or smaller variance (see Figure 14). Figure 15a shows the Isomap results from using the characteristic feature vectors of training data sets without optimization, while Figure 15b shows the results of feature vectors of training data sets after optimization by PSO. Figure 15a shows that the case of defective bearing D15, i.e., having the least severe indentation, appeared very close to the group of healthy bearings. This means that the vibration pattern of D15 was very similar to that of the healthy bearing condition, due to the small size of the defect. Thus, it is difficult to monitor the bearing integrity in the early stage, given equal weights on the IMF-based parameter vector. On the other hand, Figure 15b shows that the results from the optimally adjusted weights through PSO showing fault signals of D15, D30, D45 states and Healthy conditions were well separated.

**Figure 16:**
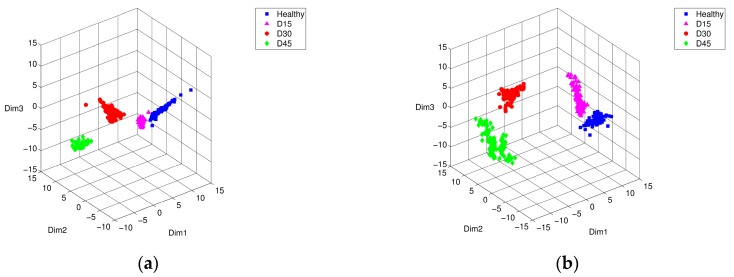
Isomap representation of testing characteristic vectors from a bearing testbed: conditions of Healthy (■), D15 (▲), D30 (●), and D45 (♦), (**a**) before, and (**b**) after PSO optimization.

Having finished the training process for choosing optimally weighted parameter vectors, we tried the test using different data sets. Again, the Isomap results of parameter vectors using the testing data sets, D15, and the healthy state are relatively close to each other. However, the Isomap results of Figure 16b using the optimized characteristic vector weights using D15, D30, and D45 signals among the testing data sets show that they are each clearly distinguishable from the others. It appears to be valid to use the optimized weights acquired from the training data sets for other arbitrary bearing data sets. This means that optimization of the weighting of the feature vectors using the PSO is effective in the experimental results. 

## 6. Conclusions

This study proposed a condition monitoring method for classifying bearing defects by constituting the damage-sensitive feature vector using EEMD in conjunction with optimized weights of the vector by a PSO algorithm. The bearing defect classifying process included both numerical simulation and validation with experimental data. Here, we suggested an optimization technique that is key to enhancing damage sensitivity. The PSO determines the best-fit parameter vectors from IMFs of EEMD, so that inner-race surface defect and rolling element defect are well separated through the dimensional reduction method of PCA and Isomap. All the bearing data were divided by training and the testing sets, to perform cross-validation of the optimized feature vector weights. For the case of experimental data collected from a real bearing system, a small size dent-type defect (D15) on the inner-race bearing showed little difference in feature vectors compared to the healthy bearing condition. However, the study found that it was possible to enhance the separation of D15 cases from the healthy condition group by optimizing the parameter weightings through PSO. Both simulation and experimental results revealed that the performance of the bearing condition classification can be improved through proper tuning of the defect-sensitive feature-vectors. Future work will include adaptation of advanced classification methods and intelligent prediction platform through modular augmented visual representation of bearing data. 

## Figures and Tables

**Table 1:** Statistical parameters of damage-sensitive feature vector: standard deviation, root mean square (RMS), kurtosis, zero-crossing rate, and entropy.

Feature	Equation
Standard Deviation	$\sqrt{E\left[{\left(x\left(t\right)-\mu \right)}^{2}\right]}$
RMS	$\sqrt{E\left[x{\left(t\right)}^{2}\right]}$
Kurtosis	$\frac{E\left[{\left(x-\mu \right)}^{4}\right]}{{\left(E\left[{\left(x-\mu \right)}^{2}\right]\right)}^{2}}$
Zero-crossing rate	$\frac{1}{T-1}\overset{T-1}{\underset{t=1}{\sum }}II\{x\left(t\right)x\left(t-1\right)<0\}$
Entropy	$\overset{n}{\underset{i=1}{\sum }}p_{i}\;\log\left(p_{i}\right)$

**Table 2:** System parameters and frequency values for generating bearing signals.

Parameter	Description	Value
$f_{r}$	Resonance frequency	800 Hz
$f_{r}$	Shaft rotating frequency	16.67 Hz
$f_{di}$	Inner-race fault frequency	82.02 Hz
$f_{do}$	Outer-race fault frequency	51.34 Hz
$f_{dr}$	Rolling element fault frequency	34.34 Hz
μ	Ratio coefficient	0.55
$F_{s}$	Sampling frequency	4 kHz
$t_{max}$	Simulation time	2 s
